# Mechanical ventilation strategies alter cardiovascular biomarkers in an infant rat model

**DOI:** 10.14814/phy2.13553

**Published:** 2018-01-22

**Authors:** Philipp Baumann, Susanne Wiegert, Francesco Greco, Sven Wellmann, Pietro L'Abate, Vincenzo Cannizzaro

**Affiliations:** ^1^ Department of Intensive Care Medicine and Neonatology University Children's Hospital of Zurich Zurich Switzerland; ^2^ Children's Research Centre University Children's Hospital of Zurich Zurich Switzerland; ^3^ Zurich Centre for Integrative Human Physiology Zurich Switzerland; ^4^ Department of Neonatology University Children's Hospital of Basel Basel Switzerland

**Keywords:** Biomarker, hyperoxia, hypoxia, positive end‐expiratory pressure

## Abstract

Mechanical ventilation (MV) is routinely used in pediatric general anesthesia and critical care, but may adversely affect the cardiocirculatory system. Biomarkers are increasingly measured to assess cardiovascular status and improve clinical treatment decision‐making. As the impact of mechanical ventilation strategies on cardiovascular biomarkers in ventilated infants is largely unknown, we conducted this retrospective study in a healthy in vivo infant rat ventilation model using 14‐days old Wistar rats. We hypothesized that 2 h of mechanical ventilation with high and low positive end‐expiratory pressure (PEEP), hyperoxemia, hypoxemia, hypercapnia, and hypocapnia would significantly impact B‐type natriuretic peptide (BNP), vascular endothelial growth factor (VEGF), and endothelin‐1 (ET‐1). We found BNP to be driven by both high (9 cmH_2_O) and low (1 cmH_2_O) PEEP compared to ventilated control animals (*P* < 0.05). VEGF concentrations were associated with high PEEP, hyperoxemia, hypoxemia, and hypocapnia (*P* < 0.05), whereas ET‐1 levels were changed only in response to hypoxemia (*P* < 0.05). In conclusion, the mode of mechanical ventilation alters plasma biomarker concentrations. Moreover, BNP and VEGF might serve as surrogate parameters for ventilation induced cardiovascular compromise and lung tissue damage. Furthermore, our data support the hypothesis, that sudden onset of hyperoxemia may trigger a quick VEGF release as a possible cellular survival reflex.

## Introduction

Invasive mechanical ventilation (MV) is one of the most frequent interventions in pediatric anesthesia and critical care (Kambas et al. 2011; Barreira et al. 2015). MV is lifesaving for the vast majority of children and has a broad acknowledged spectrum of treatment indications including surgical peri‐ and postoperative general anesthesia as well as acute respiratory and cardiovascular failure (Mehta et al. 2015). Its main therapeutic functions are to reduce or eliminate work of breathing, permit adequate oxygenation, and remove carbon dioxide. Modern concepts of invasive MV mainly involve different modes of positive pressure ventilation alongside with oxygen supply, which induce several changes to the patient's cardiovascular system (Pinsky 2005; Frazier 2008). The transition from negative pleural pressure throughout the spontaneous breath cycle to nonphysiological positive intrathoracic pressure under MV compromises venous blood return to the right atrium (Pinsky 2005; Berger et al. 2016). Furthermore, hyperinflation of alveoli and compression of small pulmonary vessels increase pulmonary vascular resistance (PVR) and right ventricular afterload leading to elevated right atrial pressure, myocardial stretch, workload, and oxygen demand (Cherpanath et al. 2016). In addition, high PVR and mechanical compression of the cardiac chambers diminish pulmonary venous return to the left atrium reducing left ventricular output (Orde et al. 2015).

Despite the risks associated with MV per se certain clinical situations require rather unusual ventilation strategies that further increase the risk for adverse effects: For example, a higher mean airway pressure (MAP) may be needed for the recruitment of closed alveoli to improve gas exchange and work of breathing. This is particularly important, as currently recommended protective ventilation strategies with low tidal volumes (*V*_T_) bear the risk of both alveolar derecruitment and hypercapnia, the latter being associated with acidosis, pulmonary vasoconstriction, and cerebral vasodilatation (Adrogué and Madias 1998a,b; Ito et al. 2003). The contrary, a low MAP and a low positive end‐expiratory pressure (PEEP) might be indicated to facilitate passive pulmonary blood flow after palliative surgery in children with univentricular hearts. In addition, high fractions of inspired oxygen (FiO_2_) are often applied for the treatment of persistent pulmonary hypertension of the newborn to decrease PVR and improve oxygenation, pulmonary blood flow, and eventually cardiac output (Lakshminrusimha et al. 2007; Kheir et al. 2013).

Hence, MV clearly affects the cardiovascular system both under physiological and pathophysiological conditions. Determination of biomarkers is increasingly used and advocated for detection, diagnosis, treatment decision‐making, and even prediction of morbidity and mortality in a variety of cardiovascular pathologies (Domico and Allen 2016; Felker et al. 2017). Though the measurement of a cardiovascular biomarker is quite easy to perform and has the potential to provide valuable additional information for optimal patient treatment, it needs to be emphasized that confounding factors such as MV have not been systematically investigated in standardized settings for the field of pediatric critical care. Moreover, an ideal biomarker should alter decision‐making, have a high sensitivity and specificity, and discriminate between the condition of interest and other confounding factors that may be especially encountered in critically ill children (Pletcher and Pignone 2011).

Standardized in vivo animal models provide a unique opportunity to investigate the influence of different ventilatory strategies on the cardiovascular system and its related plasma biomarkers. Thus, we performed this retrospective study in a healthy (non‐neonatal) infant rat ventilation model. We used remaining plasma samples from a former ventilation experiment (L'Abate et al. 2013) to evaluate effects of short term cardiovascular changes induced by high and low PEEP, hyper‐ and hypoxemia as well as hyper‐ and hypocapnia under highly standardized conditions on a set of plasma biomarkers representative for different aspects of cardiovascular compromise: B‐type natriuretic peptide (BNP) for strain of cardiomyocytes (de Bold et al. 1981; Haug et al. 1993), vascular endothelial growth factor (VEGF) for lung tissue damage and induction of angiogenesis in hypoxia (Yancopoulos et al. 2000; Choi et al. 2003), and endothelin‐1 (ET‐1) for pulmonary vasoconstriction (Fagan et al. 2001). Based on the available literature and the expected pathophysiological effects described above, we hypothesized that 2 h of positive‐pressure ventilation with different ventilator settings would have a significant impact on plasma BNP, VEGF, and ET‐1.

## Methods

### Ethical approval

The cantonal authority of Zurich approved all animal experimental protocols according to the Swiss Animal Welfare Legislation (reference number 50/2010).

### Animals

Infant Wistar rats of either sex together with their dams were supplied by Charles River Laboratories International, Inc. (Sulzfeld, Germany) at the age of ∼7 days. The animals were allowed to acclimatize for further 7 days before the experiments. Dams were kept under 12 h light‐dark cycles receiving food and water ad libitum.

### Experimental design

The animal experiments were performed between February and July 2011. The protocols for animal preparation, allocation to study groups, and MV were reported previously (L'Abate et al. 2013). Briefly, at the day of the experiment, anesthesia was induced with inhalative isoflurane followed by an intraperitoneal (IP) injection of ketamine (75 *μ*g/g body weight, BW) and xylazine (12 *μ*g/g BW) solution. A tracheostomy was conducted by insertion of a tracheal tube and the infant rats were connected to a computer‐controlled ventilator (flexiVent^®^, Scireq, Montreal, Canada) using the following baseline settings: *V*_T_ ∼8.0 mL/kg, PEEP 5 cmH_2_O, respiratory rate (RR) 90 min^−1^, and inspired oxygen fraction (FiO_2_) 0.4. Lung volume history was standardized by two lung volume recruitment maneuvers (up to 40 cmH_2_O with 9‐s ramp and 3‐s plateau) separated by a pressure‐volume (PV) maneuver (from 0 cmH_2_O to 25 cmH_2_O, 7‐s ramp and 7‐s decrease) within 2 min. Heart rate (HR) and peripheral oxygen saturation (SpO_2_) were monitored via an animal pulse oximeter (MouseOx™, STARR Life Sciences Corporation™, Oakmont, PA, USA). Body temperature was controlled and kept constant at 38°C by a thermocouple feedback rectal sensor connected to a heating mat (Physitemp Instruments, Incorporation, TCAT‐2LV Temperature Controller, Clifton, NJ, USA).

We randomly allocated the animals to the following groups. “Control” (i.e. MV with V_T_ ∼8 mL/kg, PEEP 5 cmH_2_O, FiO_2_ 0.4, RR 90 min^−1^), “PEEP 1” (PEEP 1 cmH_2_O, rest of settings unchanged) and “PEEP 9” (PEEP 9 cmH_2_O, RR 120 min^−1^, rest of settings unchanged). RR in the “PEEP 9” group was set at 120 min^−1^ in order to achieve standard blood gas values. PEEP levels were regulated by submerging the distal part of the expiratory tube into a water column. “Hypoxemia” and “hyperoxemia” were induced by FiO_2_ of 0.21 and 1.0, respectively, with the rest of ventilator settings as set at baseline. Lastly, “hypocapnia” and “hypercapnia” were induced by RR of 180 min^−1^ and 60 min^−1^, respectively, with the rest of ventilator settings unchanged. After 30 min of MV, all animals were IP injected with 0.5 mL 0.9% NaCl to avoid dehydration. Approximately 15 min before the end of the ventilation protocol, animals were IP anaesthetized with a ketamine/xylazine top‐up. The cause of death in animals used for experiments was exsanguination under full sedation. Dams were culled via exposure to carbon dioxide.

### Verification of the experimental conditions

**Figure 1:**
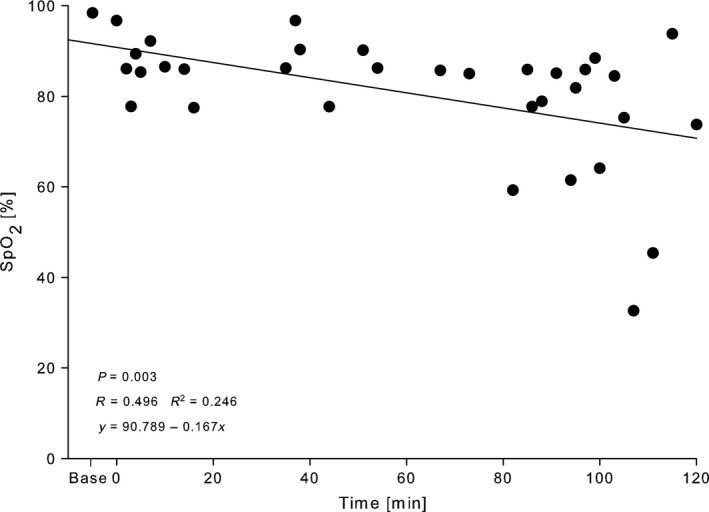
Pulse oximetric oxygen saturation (SpO_2_) of 2‐week‐old Wistar rats during mechanical ventilation with FiO_2_ 0.21 for 2 h. Data are expressed as scatter plot with regression line and corresponding equation.

Values of SpO_2_, HR and partial pressure of carbon dioxide (pCO_2_) for the experimental groups are shown in Table 1. The respective condition could be evidenced by the following mixed blood gas parameters measured at the end of the experiment: low O_2_ saturations (mean ± SD: 77.6 ± 12.6%) for hypoxemia, low pCO_2_ (3.5 ± 0.7 kPa) for hypocapnia, and elevated pCO_2_ (8.0 ± 1.4 kPa) for hypercapnia when compared to controls. In addition, the linear decrease in SpO_2_ for the hypoxemic conditions was determined throughout the ventilation protocol by peripheral pulse oximetry: 98.4 ± 0.3% at baseline, 96.8 ± 2.8% at allocation, and 73.8 ± 17.0% at the end of ventilation (Fig. 1).

**Table 1:** SpO_2_, heart rate, pCO_2_, lactate, and pH after 120 min of mechanical ventilation

Groups	SpO_2_ (%)	Heart rate (beats/min)	pCO_2_ (kPa)	Lactate (mmol/L)	pH
Control (*n* = 7)	98.5 (0.5)	292 (17)	5.2 (0.3)	0.8 (0.2)	7.42 (0.03)
PEEP 1 (*n* = 8)	98.3 (0.2)	296 (13)	5.0 (0.3)	0.9 (0.1)	7.42 (0.03)
PEEP 9 (*n* = 9)	98.6 (0.5)	284 (25)	4.9 (0.5)	1.1 (0.3)	7.41 (0.05)
Hypoxemia (*n* = 10)	**77.6 (12.6)** a	297 (17)	5.2 (0.8)	**2.0 (0.6)** a ^,^ b	7.39 (0.07)
Hyperoxia (*n* = 9)	98.9 (0.4)	291 (19)	6.1 (1.2)	**0.8 (0.2)** b	7.38 (0.06)
Hypocapnia (*n* = 10)	98.5 (0.5)	306 (18)	**3.5 (0.7)** a ^,^ b	**2.4 (1.1)** a ^,^ b	**7.54 (0.07)** a ^,^ b
Hypercapnia (*n* = 10)	98.2 (0.8)	281 (23)	**8.0 (1.4)** a ^,^ b	**0.7 (0.2)** b	**7.25 (0.08)** a ^,^ b

Data are expressed as group means ± standard deviation.

Bold highlights general statistical significance.

a Indicates statistically significant differences compared to control only.

b Indicates statistically significant differences between pairs.

### Sampling and processing of blood for biomarker and blood gas analyses

Blood sampling and blood gas analyses were performed as described by L'Abate et al. (2013). Blood was withdrawn by direct cardiac puncture after a partial laparotomy and sternotomy before disconnection from the ventilator. Blood gas analysis was conducted via a portable blood analyzer (i‐STAT^®^, Axonlab, Abbott Laboratories, IL, USA) using few drops of whole blood. The rest of the blood was collected in EDTA tubes for centrifugation at 3000*g* for 10 min. Plasma was transferred into a new EDTA tube and stored at −80°C until further analyses.

### Analysis of cardiovascular biomarkers

Plasma samples for this study were assayed between November 2014 and January 2015 in triplets in a blinded fashion according to manufacturer's instructions. BNP plasma concentrations were measured via a commercial enzyme‐linked immunosorbent assay (ELISA) kit (catalog no. 20867, Bio‐Medical Assay Co., Ltd) using a 1:10 dilution in a sample diluent provided with the kit. Determination of plasma concentrations of VEGF was performed using Rat Vascular Injury Magnetic Bead Panel 1 (RV1MAG‐26K) along with Luminex^®^ xMAP^®^ technology by EMD Millipore MILLIPLEX^®^ system. Plasma samples were diluted 1:4 in the serum matrix provided in the kit. ET‐1 plasma quantity was measured with a rat ET‐1 ELISA kit (catalog no. CSB‐E06979r, Cusabio Biotech Co.) in a 1:10 dilution in a sample diluent supplied with the kit.

### Statistical analysis

All statistical analyses were performed using the software package SigmaStat 3.5, SigmaPlot 10.0 was used for figures (Systat Software, Inc., Point Richmond, CA, USA). An unpaired *t*‐test was applied for group comparison of cardiovascular biomarkers. One‐way ANOVA was used for group comparison of HR, pCO_2_, and weight. Where appropriate, that is if group means were significantly different, Holm‐Sidak post hoc test was used for pairwise comparison of means. Transformation of data was used where suitable to ensure assumptions of normality and or equal variance. Values are reported as means ± standard deviation (SD), unless otherwise stated. Statistically significant data are additionally expressed as vertical box plots with median, 10th, 25th, 75th, and 90th percentiles. SpO_2_ progression with FiO_2_ 0.21 was formulated as scatter plot with regression line. Statistical difference was set at *P* < 0.05.

## Results

At the age of 14 days of life, animals weighed (mean) 36.2 g (SD ± 3.6 g) before being used for the ventilation experiments. The number of animals in the respective ventilation strategy is shown in Table 2.

**Table 2:** Cardiovascular biomarker concentrations in plasma after 2 h of different ventilation strategies

Group	Biomarker					
	BNP [pg/mL)		VEGF [pg/mL]		ET‐1 [pg/mL]	
	Median (IQR)	*P* value	Median (IQR)	*P* value	Median (IQR)	*P* value
Control (*n* = 7)	26.7 (22.5–34.2)	n. a.	328.0 (298.0–350.0)	n. a.	22.3 (21.3–24.1)	n. a.
PEEP1 (*n* = 8)	**38.3 (32.5**–**44.2)** a	**0.04**	480.0 (378.0–586.0)	0.10	19.9 (16.9–23.8)	0.33
PEEP9 (*n* = 9)	**43.3 (35.8**–**53.3)** a	**0.03**	**628.0 (360.0**–**762.0)** a	**0.01**	23.1 (18.5–25.9)	0.31
Hypoxemia (*n* = 10)	26.7 (18.4–29.2)	0.40	**488.0 (454.0**–**556.0)** a	**0.01**	**15.0 (11.8**–**18.4)** a	**0.03**
Hyperoxemia (*n* = 9)	33.3 (26.7–36.7)	0.17	**468.0 (382.0**–**514.0)** a	**0.03**	19.5 (14.1–22.8)	0.27
Hypocapnia (*n* = 10)	25.0 (19.2–31.7)	0.90	**488.0 (336.0**–**626.0)** a	**0.02**	n. a.	n. a.
Hypercapnia (*n* = 10)	20.0 (9.2–40.0)	0.80	300.0 (236.0–412.0)	0.37	n. a.	n. a.

Data are expressed as group medians (interquartile range, IQR). Control animals were exposed to fraction of inspired oxygen (FiO_2_) 0.4, positive end‐expiratory pressure 5 cmH_2_O (PEEP 5), and normocapnia. ET‐1 concentrations in hyper‐ and hypocapnia groups were not measured due to low sample volumes. BNP, B‐type natriuretic peptide; ET‐1, endothelin‐1; VEGF, vascular endothelial growth factor; IQR, interquartile range; n. a., not applicable.

Bold highlights statistical significance.

a *P* < 0.05 was considered as significant when compared to control.

### B‐type natriuretic peptide

**Figure 2:**
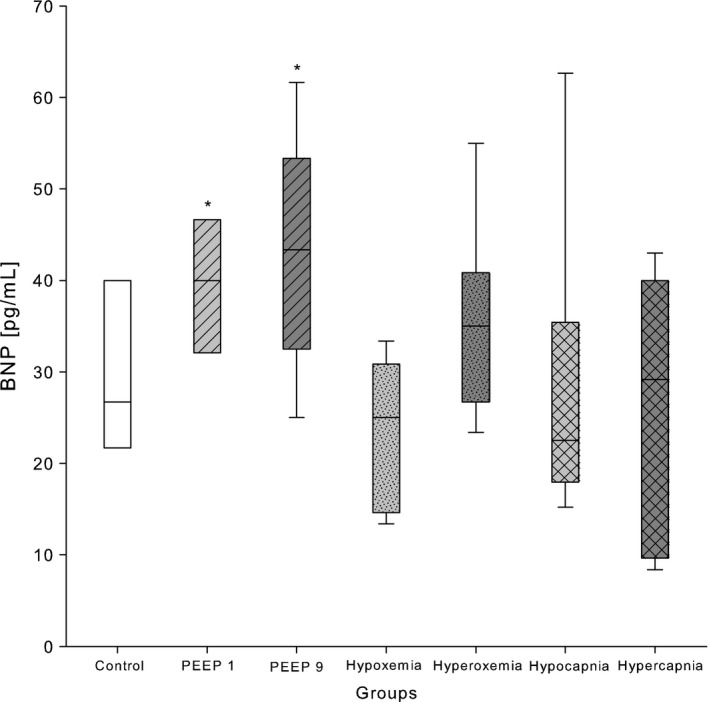
BNP concentrations in plasma of 2‐week‐old Wistar rats after exposure to different modes of mechanical ventilation for 2 h. Control: PEEP 5 cmH_2_O, FiO_2_ 0.4, RR 90 min^−1^; PEEP 1: PEEP 1 cmH_2_O; PEEP 9: PEEP 9 cmH_2_O; hypoxemia: FiO_2_ 0.21; hyperoxemia: FiO_2_ 1.0, hyperoxemia: FiO_2_ 1.0; hypocapnia: RR 180 min^−1^; and hypercapnia: RR 60 min^−1^. Data are expressed as vertical box plots with median, 10th, 25th, 75th, and 90th percentiles. *P < 0.05 was considered as significantly different when compared to control.

BNP plasma concentrations were significantly higher in both PEEP 1 and PEEP 9 groups when compared to control animals (Table 2, Fig. 2). Furthermore, no significant difference was found for hyperoxemia and hypoxemia when compared with the control group. There was also no statistically significant difference between hyper‐, hypocapnia, and control groups (Table 2).

### Vascular endothelial growth factor

**Figure 3:**
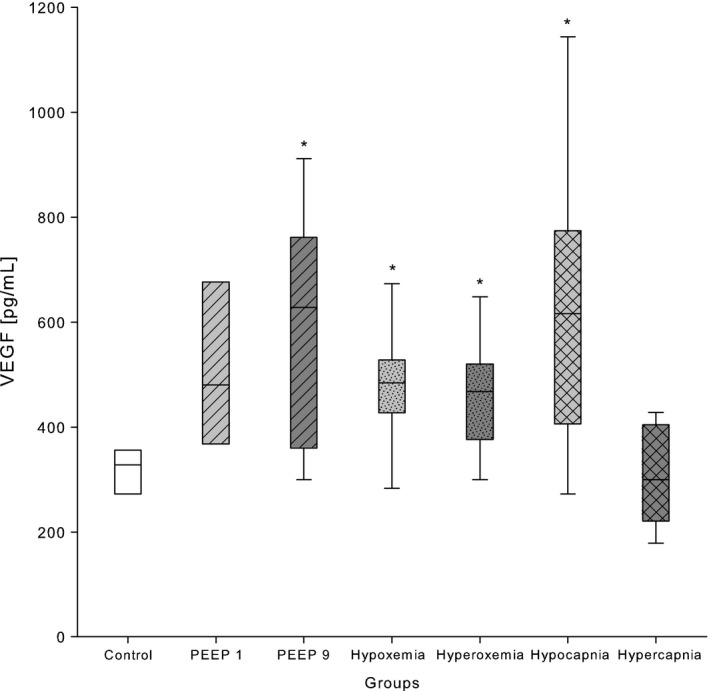
VEGF concentrations in plasma of 2‐week‐old Wistar rats after exposure to different modes of mechanical ventilation for 2 h. Control: PEEP 5 cmH_2_O, FiO_2_ 0.4, RR 90 min^−1^; PEEP 1: PEEP 1 cmH_2_O; PEEP 9: PEEP 9 cmH_2_O; hypoxemia: FiO_2_ 0.21; hyperoxemia: FiO_2_ 1.0, hyperoxemia: FiO_2_ 1.0; hypocapnia: RR 180 min^−1^; and hypercapnia: RR 60 min^−1^. Data are expressed as vertical box plots with median, 10th, 25th, 75th, and 90th percentiles. *P < 0.05 was considered as significantly different when compared to control.

VEGF protein in blood plasma was significantly elevated in the PEEP 9 group when compared to the control group, whereas the PEEP 1 group demonstrated statistically not significant higher VEGF values when compared with controls (Table 2, Fig. 3). Nevertheless, we observed significantly higher concentrations of plasma VEGF in both hyper‐ and hypoxemia groups when compared with normoxemia (Table 2, Fig. 3). Furthermore, hypocapnia induced significantly higher levels of plasma VEGF, while hypercapnia did not significantly change VEGF concentration when compared with control (Table 2, Fig. 3).

### Endothelin‐1

**Figure 4:**
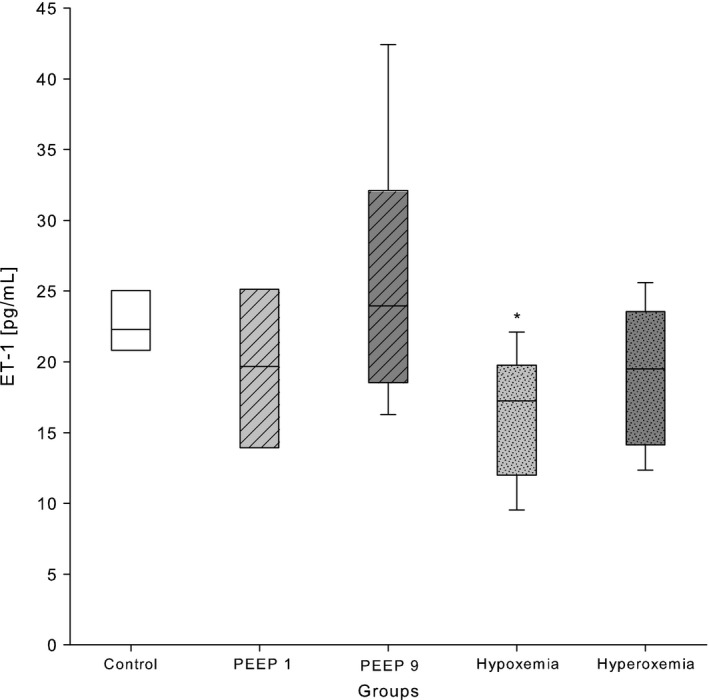
ET‐1 concentrations in plasma of 2‐week‐old Wistar rats after exposure to different modes of mechanical ventilation for 2 h. Control: PEEP 5 cmH_2_O, FiO_2_ 0.4, RR 90 min^−1^; PEEP 1: PEEP 1 cmH_2_O; PEEP 9: PEEP 9 cmH_2_O; hypoxemia: FiO_2_ 0.21; hyperoxemia: FiO_2_ 1.0, hyperoxemia: FiO_2_ 1.0; hypocapnia: RR 180 min^−1^; and hypercapnia: RR 60 min^−1^. Data are expressed as vertical box plots with median, 10th, 25th, 75th, and 90th percentiles. *P < 0.05 was considered as significantly different when compared to control.

ET‐1 plasma levels were significantly lower in the hypoxemia group when compared to normoxemia (Table 2, Fig. 4). PEEP changes and hyperoxemia did not influence ET‐1 levels. ET‐1 concentrations in hyper‐ and hypocapnia groups were not measured due to low sample volumes.

## Discussion

This retrospective study confirmed the expected impact of different mechanical ventilation strategies on plasma biomarkers. Plasma BNP was high following MV with high PEEP, but low after hypoxemic and hypercapnic ventilation. Furthermore, BNP concentration was found to be higher under low PEEP. VEGF was elevated in hypoxemia and surprisingly in hyperoxemia, too. In addition, high PEEP and hypocapnia induced unforeseen high VEGF plasma concentrations. A further unexpected finding were low ET‐1 concentrations after hypoxemia.

### B‐type natriuretic peptide

BNP is produced by the human heart in response to myocardial stretch and has diuretic, natriuretic, and vasodilating properties (de Bold et al. 1981). BNP plasma levels correlate well with left ventricular end‐diastolic pressure, pulmonary capillary wedge pressure, and right atrial pressure (Haug et al. 1993). BNP signals severity of ventricular wall stress and heart failure in children and adults (Sahingozlu et al. 2015; Xu et al. 2017). Therefore, it may serve as a proxy for ventricular strain and afterload elevation by MV. The well‐designed in vivo animal study by Kobr et al. (2011) investigated the isolated influence of injurious high *V*_T_ ventilation (*V*_T_ 10 mL/kg body weight vs. 6 mL/kg on top of PEEP 6 cm H_2_O) in healthy 7‐week‐old piglets. The investigators found a positive correlation of echocardiographically assessed left and right ventricular deterioration with plasma BNP after 12 h of ventilation. Our findings might correspond to those results and suggest that the cyclic application of high peak and mean airway pressures might impair the ventricular performance comparably to a constant application of high PEEP, although we did not perform echocardiographic measurements. We assumed that the BNP elevation in the PEEP 9 group in our study could most likely be attributed to the right ventricular afterload increase. The significant BNP elevation in the PEEP 1 group was somewhat more challenging to explain. The low PEEP should not hinder the right ventricle to eject blood, but it may be too low to avoid lung atelectasis. Several ex vivo and in vivo models have shown that low PEEP levels of 0 and 2 cm H_2_O led to larger fractions of nonventilated lung areas (Eyal et al. 1996; Luecke et al. 2003; Crespo et al. 2006). In these studies, lung collapse was accompanied by increases in PVR and PAP and consecutively by an increased burden for the right ventricle. We speculate, that this was most likely the reason for the elevated plasma concentrations of BNP in our study. Unexpectedly, we could not find an influence of hypoxemia and hypercapnia on plasma BNP concentrations. Ventilation with FiO_2_ 0.21 over 2 h caused a gradual decrease in oxygen saturation (Fig. 1) leading to SpO_2_ 78 ± 13% at the end of the experiment. This hypoxemic stimulus with a rather slow progression and a modest degree of hypoxemia after 2 h was presumably not strong enough to cause relevant pulmonary vasoconstriction and right ventricular afterload increase. Similarly, hypercapnia with a mean pCO_2_ of 8.0 kPa and a modest respiratory acidosis of pH 7.25 (Table 1) was most likely not strong enough to induce relevant pulmonary vasoconstriction with a consecutive BNP increase.

As a limitation, we did not assess ventricular function or changes in pulmonary blood flow by echocardiography. Besides that, the lack of invasive arterial pressure measurements and assessments of the PVR limited conclusions on PAP‐related BNP‐changes. Furthermore, we did not determine the BNP trajectory over time, which would have been useful to generate a time threshold when positive‐pressure ventilation becomes harmful for the right ventricle.

In summary, BNP was elevated after application of both high and low PEEP for 2 h. This was most likely due to the right ventricular compromise caused by MV and atelectasis induced right ventricular afterload elevation. BNP assessment over time in animals under MV in parallel to echocardiographic or invasive blood pressure assessments would provide further insights into the time course of the BNP elevation under the influence of MV.

### Vascular endothelial growth factor

The VEGF‐family includes five signal proteins (VEGF‐A (i.e., VEGF), VEGF‐B, VEGF‐C, VEGF‐D, and placental growth factor (PlGF)) with strong endothelial‐cell‐specific mitogenic and angiogenic properties (Yancopoulos et al. 2000). These VEGFs are found throughout both the human and rodent body with the highest presence in lung tissues (Monacci et al. 1993). PEEP exposes lung tissues to a certain degree of baseline stretch on top of which tidal volumes are added. To the best of our knowledge, no systematic investigation on the isolated influence of PEEP on VEGF has been published, so far. Nevertheless, an in vivo study with 7‐day‐old rat pups by Kroon et al. (2015) found an induction of VEGF‐gene expression following MV for 8 h with high *V*_T_ (25 mL/kg body weight) when compared to lower *V*_T_ (3.5–8.5 mL/kg). Furthermore, Choi et al. (2003) demonstrated in adult rats elevated serum VEGF concentrations after MV for 2 h with high *V*_T_ of 20 mL/kg. In light of these studies, we speculate that application of PEEP 9 cmH_2_O for 2 h was injurious enough to cause a plasma VEGF protein elevation, most likely due to the onset of lung tissue damage.

In addition, we observed high plasma VEGF protein concentrations in the hypoxemia group, as expected, according to the extended body of available evidence. The binding of HIF‐1*α* to the hypoxia response element of VEGF results in increased transcriptional activity as Ozaki et al. (1999) and Chen et al. (2012) have shown in mice models for ischemic retina and hypoxic cornea, respectively. In our study, we were able to show high plasma VEGF values after 2 h of hypoxemic MV, in line with the previously published results on hypoxia‐driven VEGF activity. However, in our study, the plasma VEGF concentration also showed a significant elevation after 2 h of hyperoxemic mechanical ventilation. This was an unexpected finding, because VEGF‐mRNA expression seems to be downregulated in hyperoxia (Alon et al. 1995; Klekamp et al. 1999). An explanation for the VEGF elevation might be the sudden impact of high oxygen concentrations on epithelial cells. Interestingly, the VEGF concentration in the epithelial lining fluid was found to increase paradoxically after hyperoxic exposure (Watkins et al. 1999; Ekekezie et al. 2003). In addition, Shenberger et al. (2007) were able to demonstrate in vitro in human lung adenocarcinoma cells and normal fibroblasts that incubation with 95% oxygen for 48 h led to a 2–3 fold increase in VEGF protein in the surrounding medium. However, VEGF‐mRNA decreased significantly in hyperoxic cells compared to normoxic controls. These findings led the group to the conclusion, that hyperoxic exposure may cause a release of VEGF protein from cell‐associated stores in the extracellular matrix to the surroundings rather than an immediate increase in mRNA‐transcription. In line with these previous studies and our results, Sato et al. (2016) could provoke a significant increase in VEGF‐C and VEGF‐D expression in mouse lung tissue by 60 h of hyperoxic exposure (FiO_2_ 0.95). A rapid initial release of preformed VEGF protein to the epithelium might happen, as already Shenberger et al. concluded, as a form of cellular survival reflex to a sudden hyperoxic exposure. The time of onset of VEGF‐release into the blood is not clear, but it may occur already within minutes after the beginning of hyperoxic exposure. Yet, Gonçalves et al. (2014) did not observe an immunohistochemical VEGF protein increase in lung tissues of newborn rat pups after 30 min of hyperoxic MV (FiO_2_ 1.0, *V*_T_ 9 mL/kg, PEEP 0 cmH_2_O). If the previously published time points of plasma VEGF measurements are hypothetically merged with our results, one can speculate on the trajectory of VEGF appearance in tissues and blood after the onset of hyperoxic exposure: the VEGF‐release from cell‐associated stores might start immediately after oxygen exposure, with VEGF protein being primarily undetectable in lung tissue at 30 min (Gonçalves et al. 2014), then being detectable and reaching significantly increased concentrations in blood plasma at 120 min, as in our study, and persistently elevated up to 48 h and more (Sato et al. 2016).

As a limitation, we did assess neither intracellular or extracellular matrix VEGF‐mRNA nor plasma VEGF protein concentration over time. Therefore, it remains unclear, if the plasma VEGF protein would have risen or fallen under continued hyperoxemic MV. A structured time‐dependent evaluation of early intracellular VEGF transcription, VEGF protein concentrations in cell‐associated stores in lung tissues and in plasma would shed further light on cellular VEGF‐reactions in case of sudden hyperoxic impacts.

Lastly, hypocapnia induced a positive VEGF response in blood. As it is known that hypocapnia causes cerebral vasoconstriction resulting in reduced cerebral blood flow and reduced cerebral blood volume (Ito et al. 2003), it is suggestive, that the consecutively reduced cerebral oxygen delivery caused cerebral tissue hypoxia. This might be reflected by the significantly elevated plasma lactate in this group (mean 2.4 mmol (±SD 1.1), Table 1) compared to ventilated control animals, while the peripherally measured oxygen saturation remained perfectly stable in the normal range at 98.5% (±0.5). As to the best of our knowledge, no studies were published so far associating hypocapnia with VEGF. However, hypocapnia has been identified as an independent risk factor for cerebral leukomalacia in the preterm birth child and was linked to poor neurodevelopmental outcome (Resch et al. 2012). Therefore, we might speculate that an elevated plasma VEGF during hypocapnic episodes in ventilated children could be used as an early proxy for cerebral hypoperfusion and cerebral tissue hypoxia. This conclusion is limited by the lack of long‐term neurological and histological follow‐up in our study leaving the developmental relevance of this finding undetermined.

In summary, we could show a significantly elevated VEGF in plasma after 2 h of MV with PEEP 9 cmH_2_O, hyper‐ and hypoxemia, and with hypocapnia. Whether and when VEGF is released after sudden hyperoxemic exposure remains the matter of future research.

### Endothelin‐1

ET‐1 is an endogenous peptide with strong vasoactive properties involved in various lung disorders such as pulmonary hypertension, acute respiratory failure and development of bronchopulmonary dysplasia (Fagan et al. 2001; Kambas et al. 2011). A broad spectrum of studies have shown hypoxia to be a strong stimulus for the HIF‐1*α* mediated ET‐1 transcription (Hu et al. 1998; Paradis et al. 2015). However, in our study, ET‐1 was significantly lower after 120 min of hypoxemic ventilation, which is in contrast to the generally assumed stimulating effect of hypoxia on plasma ET‐1. Nevertheless, our results do not have to be necessarily contradictory to the previously published studies when taking into account the dose‐dependency of ET‐1 protein appearance in blood after onset of hypoxia. Shirakami et al. (1991) exposed rats to FiO_2_ 0.1 and observed an increase in ET‐1 protein in lungs and peripheral plasma after 60 min of exposure. The effect appeared significantly earlier (already after 10 min) and stronger, if the rats were exposed to FiO_2_ 0.05. Helset et al. (1995) found increased ET‐1 protein levels in the perfusate of isolated rat lungs even earlier, that is after 5 min exposure to FiO_2_ 0.02. FiO_2_ 0.21 in our study was not sufficient to maintain adequate oxygen saturation over 120 min of ventilation (SpO_2_ ~78% after 120 min). This is most likely due to the gradual de‐recruitment of alveoli under standard ventilation over the experimental time leading to an insufficient oxygenation. This form of hypoxemic stimulus probably provoked a slower ET‐1 release than found in the more aggressive approaches used by the groups of Shirakami and Helset. Another contributing factor may be the increase in ET‐1‐receptor density under hypoxic conditions as shown by Li et al. (1994a,b) and Takahashi et al. (2001). The ET_B_ receptor subtype concentration was found to be relatively higher than ET_A_ in pulmonary arteries after hypoxic exposure, which is suggestive for ET_B_ to be the primary clearance site (Li et al. 1994b; Kelland et al. 2010). Up to date, the earliest assessment of the ET‐1 receptor‐mRNA in hypoxic rat lungs was performed after 12 h of hypoxic exposure, showing a significant increase in receptor abundance. A potential early increase in ET‐1 receptor transcriptional and translational activity within the first hours of hypoxic exposure may cause an increased binding of circulatory ET‐1 protein to more available binding sites. This increased ET‐1 protein clearance from the blood stream might have been a second factor contributing to the low ET‐1 protein level found in hypoxemia in our study.

As limitations, we did not perform serial ET‐1 protein measurements and did not assess the ET‐1‐receptor density, which would have been useful for further conclusions on the ET‐1 protein‐receptor‐interaction in hypoxemic ventilation.

In summary, we can speculate, that ET‐1 was low following hypoxemic ventilation most likely due to the combination of a hypoxic stimulus that developed gradually over 120 min and an increase in ET‐1‐receptor density.

### Limitations

Besides the limitations mentioned above for each biomarker, our study was limited by the relatively short period of ventilation (2 h) and by the biomarker examination at a single time point. Furthermore, comparisons to echocardiography, invasive blood pressure and flow measurements, and histology would have been useful to relate objectively measured cardiovascular compromise to biomarker findings. However, as the ventilatory experiments presented here were performed in a highly standardized fashion, we deem the altered biomarkers as being valid surrogates for organ specific compromise.

One important limitation for the biomarker measurements in this study is the potential protein breakdown over storage time. The plasma samples were stored between the animal experiments and the VEGF measurements at −80°C for a period of maximum 4 years 9 months. VEGF is known to degrade significantly in serum, even at very low storage temperatures (−75°C) (Kisand et al. 2011), but not in plasma at −80°C (Hetland et al. 2008). However, as the longest published period of VEGF plasma storage at −80°C spans 2 years (Hetland et al. 2008) a partial degradation of VEGF over the storage time could be possible. For ET‐1 and BNP time until assay was maximum 4 years 11 months. Long‐term stability for ET‐1 storage at −80°C was not assessed until to date, but Perez et al. (2016) found ET‐1 to be stable in plasma for 9 months when stored at −70°C. We assume, that storage at −80°C did not seriously affect ET‐1 plasma levels, but a bias over time could not be excluded. Plasma BNP levels were found to decrease by 1% per month when stored at −80°C, but low levels (<100 pg/mL) were relatively stable over a maximum published period of 1 year (Pereira et al. 2008). As the evidence for long‐term stability is limited for the three biomarkers we could not exclude a degradation effect over time as potential bias on absolute plasma values.

Last, as systematic data on the influence of mechanical ventilation on biomarkers in human pediatric patients is scarce, the transfer of conclusions from animal studies to human beings has to be done, as it is necessary for any model, with caution. Future ventilatory studies in human beings may reveal significant differences to animal models.

## Conclusion

Our selected MV strategies compromised the pulmonary and systemic circulation and induced lung tissue damage as reflected by cardiovascular biomarker elevations in plasma. BNP might have been driven by right ventricular afterload elevation induced by inadequate PEEP levels, whereas VEGF might have been high in ventilation strategies with excessive mechanical lung stretch, lung injury, and reduced cerebral perfusion. Furthermore, our data support the hypothesis from previous studies that high plasma VEGF levels in hyperoxia seem to reflect a quick cellular release mechanism possibly important for cellular survival. ET‐1 was unexpectedly low in hypoxemia most likely due to a combination of a rather mild hypoxemic stimulus and a presumed increase in ET‐1‐receptor density that might have caused early blood stream clearance. The influence of positive pressure ventilation and oxygen application has to be taken into account for the clinical interpretation of cardiovascular biomarkers.

## Conflict of Interest

None.
